# Research on the Pose Error Compensation Technology for the Mass and Centroid Measurement of Large-Sized Aircraft Based on Kinematics

**DOI:** 10.3390/s23020701

**Published:** 2023-01-07

**Authors:** Hang Yu, Xiaolin Zhang, Zanqin Wang, Wenyan Tang, Jun Wang

**Affiliations:** School of Instrumentation Science and Engineering, Harbin Institute of Technology, Harbin 150001, China

**Keywords:** mass, centroid, load cell, mechanical structure, modeling

## Abstract

The accurate measurement of mass and centroid is indispensable for accurate control of aircraft. In order to eliminate the influence of assembly error and product pose error on the measurement results, a multi-station measurement idea that can self-compensate for the geometric parameter error is proposed in this paper based on. At the same time, the kinematic model of the mechanical structure of the measurement equipment is established to prove the effectiveness of the structure in error compensation theoretically, and the experimental verification of the standard part and aircraft is carried out. The test results show that the measurement results using the idea of the multi-station compensation measurement are significantly better than those of the more common methods, with the mass measurement accuracy of 0.03% and the centroid error within ±0.15 mm, meeting the requirement of high precision measurement for the mass properties.

## 1. Introduction

The accurate measurement of the aircraft mass and centroid is a prerequisite for the precise control of aircraft. The mass of the aircraft reflects its carrying capacity, and the accuracy of the aircraft mass measurement directly affects the correctness of its force analysis and dynamics modeling [1]. The position of the aircraft centroid determines the origin of its trajectory-related coordinate system; for example, the origin of the ballistic, projectile body and velocity coordinate systems are all selected at the centroid of the aircraft. Deviations of the centroid measurement will lead to inaccurate dynamics modeling and the scalar equation of kinematics of the aircraft, thus affecting the final flight trajectory [2]. With the rapid development of aerospace technology in recent years, the aircraft structure has already broken through the limitations of small and medium-sized rotating bodies. Higher accuracy requirements in trajectory and attitude and more difficult control of the new aircraft have been raised for its special shape structure, which has also put forward higher requirements on the accuracy of mass and centroid measurement [3].

There are two main methods for determining the mass and centroid parameters; one is the computer simulation analysis method [4], and the other is the experimental measurement method [5]. Since the workload of simulating the real situation of a large-sized and heterogeneous structure is huge and the simulation analysis cannot completely simulate the real situation, the simulation analysis results are often just a reference, which is needed to verify each other experimentally. A variety of experimental test methods, such as multi-point weighing, a compound pendulum, a three-wire pendulum, and visual measurement, can be used as the experimental measurement. Na established a measurement unified model of the multi-configuration planetary rover based on the measurement characteristics of the static balance equation and multi-point weighing method [6]. Dario proposed a method based on the class of suspension techniques and dual-axis inclinometer readings for determining the center of gravity of a small spacecraft [7]. Oliveira estimated the 2D coordinates of the projection of center of gravity (CG) of an object by constructing a reaction board mounted on three supports instrumented with FBGs and applying the equilibrium conditions of a rigid body and proper calibration procedures [8]. Wang presented an experimental method to measure the gravity center of an UAV by using the projection method and the compound-pendulum device [9].

The measurement methods above have been developed for a long time and have been able to achieve considerable accuracy when the measured parts are of moderate size and regular shape, meeting the general measurement requirements [10]. However, the following problems still exist for the mass and centroid parameters measurement of large-sized aircraft which is difficult to install by manpower in terms of size and mass, and corresponding hoisting equipment is required to achieve the effect. Firstly, these measurement methods involve a variety of problems in the actual operation process, such as the need for multiple clamping, complex clamping processes, low efficiency, and the introduction of large positioning errors. Secondly, in the process of centroid measurement of the measured target, there is often a certain geometric error in the process of mounting the tooling, which leads to the actual position of the measured object not matching the theoretical situation, resulting in systematic errors in the centroid measurement result. When the measured object is a small or medium-sized warhead or a part of the cabin of a large-sized aircraft, the measurement system structure is simple, and the measurement accuracy can still be guaranteed even if the above-mentioned influencing factors are ignored [3]. However, as the size of the aircraft increases and the shape diversifies, any small assembly error will lead to the deviation of the centroid measurement value from the true value being magnified several times, which seriously restricts the further improvement of the mass and centroid parameters measurement accuracy of large-sized aircraft. Meanwhile, the research on the influence and compensation of product pose error on the measurement results is also rare. Finally, in practical applications, due to the limitation of the versatility of the measurement equipment, in order to meet the measurement requirements of different models of aircraft, it is necessary to redesign and process for individual models of aircraft, which causes great production costs [11].

In order to solve the above problems, Wang Meibao used the multi-point weighing method and combined it with the idea of flexible measurement to develop the mass and centroid test equipment suitable for large-sized aircraft [11,12]. The device uses two subsystems, and the relative position between them can be adjusted according to the size of the measured product to be compatible with a variety of models of measured products. However, the equipment only applies to rotary aircraft, and it cannot be used to measure the centroid of large-sized aircraft with an irregular shape. Additionally, it does not have the compensation function for the product attitude error. Wang Chao [10,13] researched measurement attitude error correction technology based on kinematics, considered the situation that the actual attitude of the measured product does not coincide with the theoretical attitude, analyzed the influence of the measured product attitude error on the measurement results, modeled the mechanical structure of the measurement system while calibrating its geometric parameters, and established a calibration equation based on the attitude error. However, in this process, each step needs to reflect the actual posture of the object under test, relying on the laser tracker, which greatly increases the difficulty of use and prolongs the testing time. Additionally, decoupling is required when performing error compensation, which increases the difficulty of obtaining accurate centroid data.

In view of the above situation, a multi-station measurement idea which can compensate for the pose error is proposed based on the equipment described in [14], After the initial calibration, the conversion of measurement results into the measured object’s own coordinate system without a laser tracker is realized by the homogeneous coordinate transformation matrix based on the kinematic concept. At the same time, this function can also effectively suppress product pose errors caused by assembly errors introduced by the measurement system or the object under test. In order to verify the compensation effect of the multi-station transfer device on the measurement of the product centroid, the corresponding kinematic error model is established for the mass and centroid measurement system, and the quantitative analysis of the measurement error compensation effect of the device is carried out from the theoretical level.

This paper introduces the basic principles of the mass and centroid measurement and innovatively proposes a multi-station measurement idea that can self-compensate the measurement results. A multi-station switching device is then designed, which can improve the versatility of the measurement system while compensating for pose errors. Meanwhile, it defines different coordinate systems according to the structure of the measurement system, and it gives the coordinate conversion method while establishing a general kinematic error model and proving the feasibility of the idea feasibility. Finally, the reliability of the measurement system is verified by testing on the standard part and the large-sized aircraft.

## 2. Materials and Methods

### 2.1. Theoretical Basis

**Figure 1:**
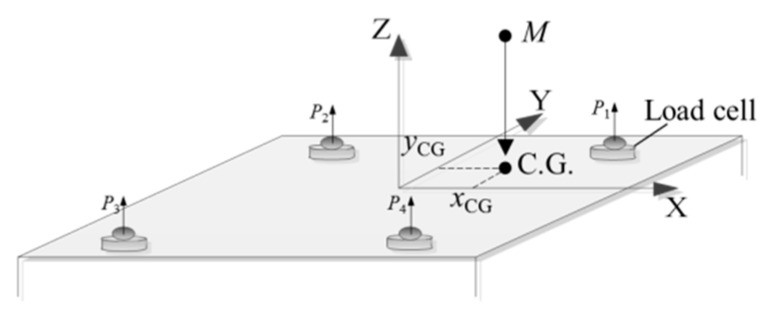
The principle diagram of the mass and centroid measurement.

As shown in Figure 1, the measurement system consists of four load cells [15].

Before the measurement, the four load cells are adjusted to the same height. Record the output value of the load cell in the no-load state and the load state, respectively, noted as *P*_1*i*_ and *P*_2*i*_ [14], namely:

(1) $$M_{g}=P_{11}+P_{12}+P_{13}+P_{14}$$

(2) $$M_{z}=P_{21}+P_{22}+P_{23}+P_{24}$$

where *M_g_* is the equipment work parts mass, and *M_z_* is the total mass of the equipment tooling and the measured object.

The mass of the object under test *M_C_* can be expressed as:

(3) $$M_{c}=M_{z}-M_{g}$$

The theoretical 2D coordinates of the centroid of the measured object in the sensors’ coordinate system can be measured through the principle of moment balance, combined with the position information and output data of the load cell.

(4) $$x_{CG}=\frac{\Delta P_{1}x_{s1}+\Delta P_{2}x_{s2}+\Delta P_{3}x_{s3}+\Delta P_{4}x_{s4}}{\Delta P_{1}+\Delta P_{2}+\Delta P_{3}+\Delta P_{4}}\;y_{CG}=\frac{\Delta P_{1}y_{s1}+\Delta P_{2}y_{s2}+\Delta P_{3}y_{s3}+\Delta P_{4}y_{s4}}{\Delta P_{1}+\Delta P_{2}+\Delta P_{3}+\Delta P_{4}}$$

where *x_si_*, *y_si_* are the coordinates of the *i*th cell in the *XOY* plane of the coordinate system where it is located, and Δ*P_i_* is the difference between the measured values before and after the *i*th cell loads the measured object, denoted as Δ*P_i_* = *P*_2*i*_ − *P*_1*i*_ [14].

Then, the coordinates are converted to the object’s own coordinate system, so that the 2D centroid coordinates can be obtained from a single measurement. Similarly, the coordinates of the other direction are measured by rotating the object accordingly.

### 2.2. Design of Multi-Station Measurement

A multi-station measurement idea that can self-compensate the results is proposed on the basis of the mass and centroid measurement system introduced in the paper [14]. The idea is realized by the multi-station switching device in [14].

**Figure 2:**
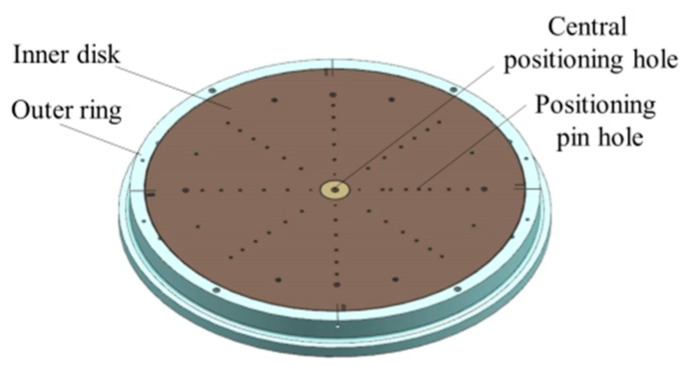
3D model of the multi-station switching device.

As shown in Figure 2, the inner disk can be rotated 360° around the central positioning hole and is positioned and locked by quadrant positioning pin holes at the boundary positions of four quadrants (noted as I, II, III, and IV), respectively.

**Figure 3:**
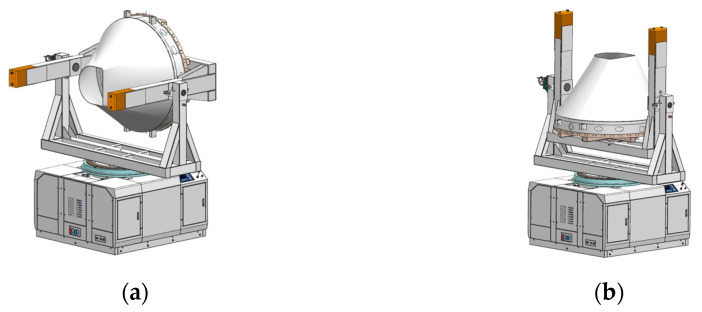
(**a**) Overall effect of the equipment—horizontal state; (**b**) overall effect of the equipment—vertical state.

Furthermore, in the measurement process, as shown in Figure 3, the multi-station switching device is connected to the measurement tooling or product through the center positioning hole and tooling positioning pin holes, which play the roles of positioning and switching between the measuring tool (or the object to be measured) and the measurement table, providing an expanded interface for the measured objects of different sizes and their matching tooling, and providing the measurement function of horizontal and vertical positions for the measured object.

In the process of measurement, firstly, only the matching tooling of the measured object is installed, and then the inner disk of the multi-station switching device is rotated to the I, II, II, and IV quadrant positions, respectively, and the measuring equipment is operated to record the output data of the four load cells in the no-load state in the four positions, respectively. Secondly, the measured object and the matching tooling are installed, and the operation of the steps above is repeated. Then, the output values of the load cells in the four positions will be recorded, which will be combined with the coordinates of the load cells in the coordinate system to obtain the centroid coordinates of each posture in the corresponding coordinate system by Equation (4). Finally, convert the results to the coordinate system of the measured object and calculate the average value to obtain the 2D centroid coordinates in the current pose.

### 2.3. Pose Errors Analysis

#### 2.3.1. Homogeneous Coordinate Transformation Matrix

The coordinates of the point *P* in the coordinate system *U* are (*^U^P_x_*, *^U^P_y_*, *^U^P_z_*) and in the other coordinate system *V* are (*^V^P_x_*, *^V^P_y_*, *^V^P_z_*), then:

(5) {PxU=PxV⋅nx+PyV⋅ox+PzV⋅ax+pxPyU=PxV⋅ny+PyV⋅oy+PzV⋅ay+pyPzU=PxV⋅nz+PyV⋅oz+PzV⋅az+pz

where *P_x_*, *P_y_*, *P_z_* are coordinates of the origin of the coordinate system *V* in the coordinate system *U*; *n_x_*, *n_y_*, *n_z_* are three direction cosines of the ***X****_v_* axis of the coordinate system *V* onto the coordinate system *U*; *o_x_*, *o_y_*, *o_z_* are three direction cosines of the ***Y****_v_* axis of the coordinate system *V* onto the coordinate system *U*; *a_x_*, *a_y_*, *a_z_* are three direction cosines of the ***Z****_v_* axis of the coordinate system *V* onto the coordinate system *U*.

Equation (5) can be written in the following form:

(6) PU=TUV⋅PV

where

PU=[PxUPyUPzU1]T,PV=[PxVPyVPzV1]TTUV=[nxoxaxpxnyoyaypynzozazpz0001]=[noap0001]=[RUVp0I]=Trans(px,py,pz)⋅Rot(k,ε)

TUV is the homogeneous transformation matrix of the coordinate system *U* to the coordinate system *V*; ***Trans***(*p_x_*, *p_y_*, *p_z_*) is the translation homogeneous coordinate transformation matrix; ***p*** = [*p_x_ p_y_ p_z_*]*^T^* is the translation vector, representing the displacement vector from the origin of the coordinate system *U* to the origin of the coordinate system *V*; RUV = [***n o a***] is the rotation transformation matrix, representing the rotation matrix of the coordinate system *U* turning to the coordinate system *V* consistently; ***Rot***(***k***, *ε*) is the rotation homogeneous coordinate transformation matrix; ***k*** is the unit vector in the direction of the rotation axis, *ε* is the angle of rotation around ***k***.

Clearly, the rotation homogeneous coordinate transformation matrix of the coordinate system *U* rotated *ε_x_* around the ***X***_U_ axis at the origin can be expressed as:

(7) $$\mathrm{Rot}(X_{U},\varepsilon_{x})=\left[\begin{array}{cccc} \cos0{}^{\circ} & \cos90{}^{\circ} & \cos90{}^{\circ} & 0\\ \cos90{}^{\circ} & \cos\varepsilon_{x} & \cos(90{}^{\circ}+\varepsilon_{x}) & 0\\ \cos90{}^{\circ} & \cos(90{}^{\circ}-\varepsilon_{x}) & \cos\varepsilon_{x} & 0\\ 0 & 0 & 0 & 1 \end{array}\right]=\left[\begin{array}{cccc} 1 & 0 & 0 & 0\\ 0 & \cos\varepsilon_{x} & -\sin\varepsilon_{x} & 0\\ 0 & \sin\varepsilon_{x} & \cos\varepsilon_{x} & 0\\ 0 & 0 & 0 & 1 \end{array}\right]$$

Similarly,

(8) $$\mathrm{Rot}(Y_{U},\varepsilon_{y})=\left[\begin{array}{cccc} \cos\varepsilon_{y} & 0 & \sin\varepsilon_{y} & 0\\ 0 & 1 & 0 & 0\\ -\sin\varepsilon_{y} & 0 & \cos\varepsilon_{y} & 0\\ 0 & 0 & 0 & 1 \end{array}\right]$$

(9) $$\mathrm{Rot}(Z_{U},\varepsilon_{z})=\left[\begin{array}{cccc} \cos\varepsilon_{z} & -\sin\varepsilon_{z} & 0 & 0\\ \sin\varepsilon_{z} & \cos\varepsilon_{z} & 0 & 0\\ 0 & 0 & 1 & 0\\ 0 & 0 & 0 & 1 \end{array}\right]$$

**Figure 4:**
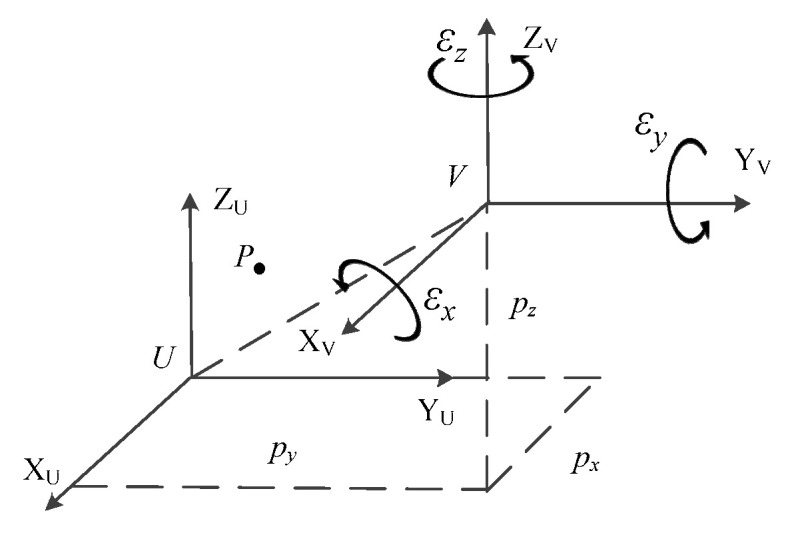
Coordinate transformation of coordinate system.

As shown in Figure 4, if the coordinate system *U* is first rotated by *ε_x_*, *ε_y_* and *ε_z_* around the ***X***_U_, ***Y***_U_ and ***Z***_U_ axes, respectively, and then translated by *p_x_*, *p_y_* and *p_z_* along the ***X***_U_, ***Y***_U_ and ***Z***_U_ axes, respectively, the homogeneous coordinate transformation matrix characterizing the rotation and translation transformation above from *U* to the new coordinate system *V* is:

(10) TUV=Trans(px,py,pz)⋅Rot(ZU,εz)⋅Rot(YU,εy)⋅Rot(XU,εx)=[cosεycosεzsinεxsinεycosεz−cosεxsinεzcosεxsinεycosεz+sinεxsinεzpxcosεysinεzsinεxsinεysinεz+cosεxcosεzcosεxsinεysinεz−sinεxcosεzpy−sinεysinεxcosεycosεxcosεypz0001]

When the rotation angles *ε_x_*, *ε_y_* and *ε_z_* are very small:

(11) $$\sin\varepsilon_{x}\approx \varepsilon_{x};\;\sin\varepsilon_{y}\approx \varepsilon_{y};\;\sin\varepsilon_{z}\approx \varepsilon_{z};\;\cos\varepsilon_{x}\approx 1;\;\cos\varepsilon_{y}\approx 1;\;\cos\varepsilon_{z}\approx 1$$

When the translational displacement is very small, *δ_x_*, *δ_y_* and *δ_z_* are used to represent *p*_x_, *p*_y_ and *p*_z_, respectively. Clearly, the micro-values above the second order can be neglected, and the homogeneous coordinate transformation matrix can be expressed as:

(12) TUV=[1−εzεyδxεz1−εxδy−εyεx1δz0001]

#### 2.3.2. Coordinate Systems Definition

**Figure 5:**
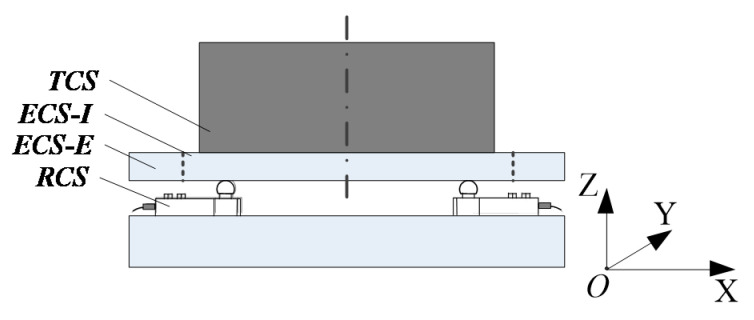
The method of establishing the coordinate systems.

The multi-station switching device is compatible with different aircraft through the tooling in order to complete their mass and centroid measurements, which would constitute a variety of different mechanical structures. For ease of expression and analysis, take the example of the measuring equipment with a standard part mounted separately via positioning pins, as shown in Figure 5. The mechanical structure of the equipment is a tandem type structure, which establishes the reference coordinate system (RCS), the target coordinate system (TCS), and the coordinate system based on the outer ring and inner disk of the multi-station switching device (ECS-E External of Equipment Coordinate System) and (ECS-I Internal of Equipment Coordinate System).

In the ideal (error-free) case, the kinematic model of the coordinates *^T^**CG*** of the centroid *^R^**CG*** measured in the product coordinate system can be expressed as:

(13) CTGi=TEITi⋅TEEEIi⋅TREEi⋅CRG

where:

TREEi=[100a0010b0001c00001],TEEEIi=[cosεz−sinεz00sinεzcosεz0000100001],TEITi=[1000010000100001]

#### 2.3.3. Establishment of the Error Model

In the actual measurement, the measurement table needs to be raised or lowered to complete the loading of the measured object, in which the ECS-E has three displacement error components *δ*_x_(*EE*), *δ*_y_(*EE*), *δ*_z_(*EE*) and three angle error components *ε*_x_(*EE*), *ε*_y_(*EE*), *ε*_z_(*EE*) with respect to the RCS. The transformation matrix of the ECS-E with respect to the RCS is:

(14) TREEe=[1−εz(EE)εy(EE)a0+δx(EE)εz(EE)1−εx(EE)b0+δy(EE)−εy(EE)εx(EE)1c0+δz(EE)0001]

There is a systematic error Δ*ε* in the rotational positioning of the inner and outer disks of the multi-station switching device. Then, the transformation matrix of the ECS-I with respect to the ECS-E is:

(15) TEEEIe=[cosεzcosΔε−sinεzsinΔε−sinεzcosΔε−cosεzsinΔε00sinεzcosΔε+cosεzsinΔεcosεzcosΔε−sinεzsinΔε0000100001]

The assembly error between the TCS and the ECS-I includes three displacement error components *δ*_x_(*T*), *δ*_y_(*T*), *δ*_z_(*T*) and three angle error components *ε*_x_(*T*), *ε*_y_(*T*), *ε*_z_(*T*). The transformation matrix of the coordinate system TCS with respect to the ECS-I is:

(16) TEITe=[1−εz(T)εy(T)δx(T)εz(T)1−εx(T)δy(T)−εy(T)εx(T)1δz(T)0001]

In the ideal case, the actual centroid coordinates of the measured object should coincide with the theoretical coordinates in space. However, in the actual case, the two are separated in space due to the error of the measured object’s pose.

Take the inner disk turned to I position (*ε*_z_ = 0°) as an example in the ideal condition. From Equation (13), the transformation matrix of the TCS with respect to the RCS is:

(17) TRTi=TEITi⋅TEEEIi⋅TREEi=[100a0010b0001c00001]

Assuming that the coordinates of the 2D centroid of the measured object in RCS are *^R^**CG*** (*x*_I*r*_,*y*_I*r*_,0). Then, ideally, the coordinates of the measured object centroid in TCS are:

(18) CTGi=TRTi⋅CRG=TRTi⋅[xIryIr01]T

However, in the actual case, considering the influence of the object’s pose error, the transformation matrix of the TCS with respect to the RCS is:

(19) TRTe=TEITe⋅TEEEIe⋅TREEe=[1−εz(E)⋅(Δε+εz(T))−εy(E)⋅εy(I)−Δε⋅εz(T)Δε+εz(T)+εy(E)⋅εx(T)−εz(E)⋅(Δε⋅εz(T)−1)Δε⋅εx(T)−εy(T)−εy(E)+εz(E)⋅(εx(T)+Δε⋅εy(T))0εx(E)⋅εy(T)−εz(T)−Δε+εz(E)⋅(Δε⋅εz(T)−1)1−εz(E)⋅(Δε+εz(T))−εx(E)⋅εx(T)−Δε⋅εz(T)εx(E)+εx(T)+Δε⋅εy(T)+εz(E)⋅(εy(T)−Δε⋅εx(T))0εy(T)+εx(E)⋅(Δε+εz(T))−εy(E)⋅(Δε⋅εz(T)−1)εy(E)⋅(Δε+εz(T))−εx(T)+εX(E)⋅(Δε⋅εz(T)−1)1−εy(E)⋅(εy(T)−Δε⋅εx(T))−εx(E)⋅(εx(T)+Δε⋅εy(T))0 δx(T)−(b1+δy(E))⋅(Δε+εz(T))+εy(T)⋅(c1+δz(E))−(a1+δx(E))⋅(Δε⋅εz(T)−1)δy(T)+(a1+δx(E))⋅(Δε+εz(T))−εx(T)⋅(c1+δz(E))−(b1+δy(E))⋅(Δε⋅εz(T)−1)c1+δz(E)+δz(I)−(a1+δx(E))⋅(δy(T)−Δε⋅εx(T))+(b1+δy(E))⋅(εx(T)+Δε⋅εy(T))1]

The actual coordinates of the centroid of the measured object in its own coordinate system are:

(20) CTGe=TRTe⋅CRG=TRTe⋅[xIryIr01]T

### 2.4. Self-Compensation Effect Analysis

The inner disk of the multi-station switching device is rotated to the positions I (*ε*_z_ = 0°), II (*ε*_z_ = 90°), III (*ε*_z_ = 180°), and IV (*ε*_z_ = 270°). Moreover, the coordinates of the object centroid (*^R^**CG***_I_, *^R^**CG***_II_, *^R^**CG***_III_, *^R^**CG***_IV_) in the current state are measured in the RCS. Then, the coordinates are converted into the TCS. The object centroid errors in the TCS with and without the product pose errors are sorted out in (21).

(21) {ΔCTGI=CTGIe−CTGIi=TRTIe⋅CRGI−TRTIi⋅CRGIΔCTGII=CTGIIe−CTGIIi=TRTIIe⋅CRGII−TRTIIi⋅CRGIIΔCTGIII=CTGIIIe−CTGIIIi=TRTIIIe⋅CRGIII−TRTIIIi⋅CRGIIIΔCTGIV=CTGIVe−CTGIVi=TRTIVe⋅CRGIV−TRTIVi⋅CRGIV

Bringing Equations (13), (17)–(20) into (21) and neglecting the micro-values above the second order, the expression of the mean value of the errors is:

(22) ΔCTG=14(ΔCTGI+ΔCTGII+ΔCTGIII+ΔCTGIV)=[δx(T)+c1⋅εy(T)δx(T)−c1⋅εx(T)c1+δz(E)+δz(I)−14⋅∑x⋅εy(E)+14⋅∑y⋅εx(E)1]

Since the measured amount is the 2D centroid of the measured object, only the first two items in Equation (22) need to be considered, and it can be seen that the 2D centroid error does not contain random variables at this time, which can be regarded as systematic error and compensated by calibration experimentation. Comparing with Equation (19), it can be seen that the measurement results of the object centroid using the multi-station switching device for multi-station measurement can greatly reduce the measurement errors and pose errors introduced by the assembly.

## 3. Results

### 3.1. Calibration Result

The equipment and the experimental process refer to the cite [14].

The test items and the measurement accuracies are given in Table 1.

**Figure 6:**
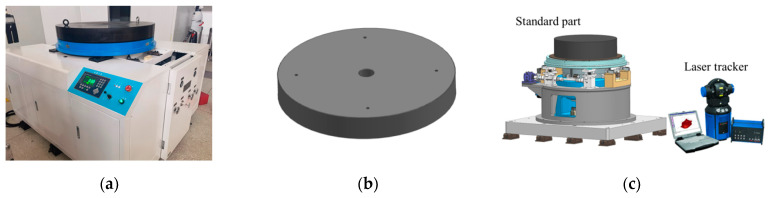
(**a**) Physical picture of the measurement system; (**b**) 3D model of standard parts; (**c**) standard sample mass centroid measurement diagram.

In order to verify that the multi-station switching device can compensate for the errors introduced by the assembly and the measured product pose errors in the mass and centroid measurement of large-sized aircraft, the standard part was tested first, as shown in Figure 6. The mass and center of gravity of the standard part have been calibrated by the metrology institution. The mass is 883.495 kg, and the center of gravity coincides with the centroid. The standard part is a pie-type structure, which can be installed and positioned with the multi-station switching device through positioning pins.

In the test process, the standard part was placed on the measuring table. First, record the measurement results of the mass and centroid of the inner disk of the multi-station switching device in position I, recorded as *M*_I_ and *CG*_I_. Second, rotate the multi-station switching device to position I, II, III, and IV, then the average of the results of the four positions of the mass and centroid is calculated and recorded as *M*_avg_ and *CG*_avg_. Finally, the geometric center position of the plane on the standard part was measured by the laser tracker and compared with the *CG*_avg_, as shown in Figure 6c.

**Figure 7:**
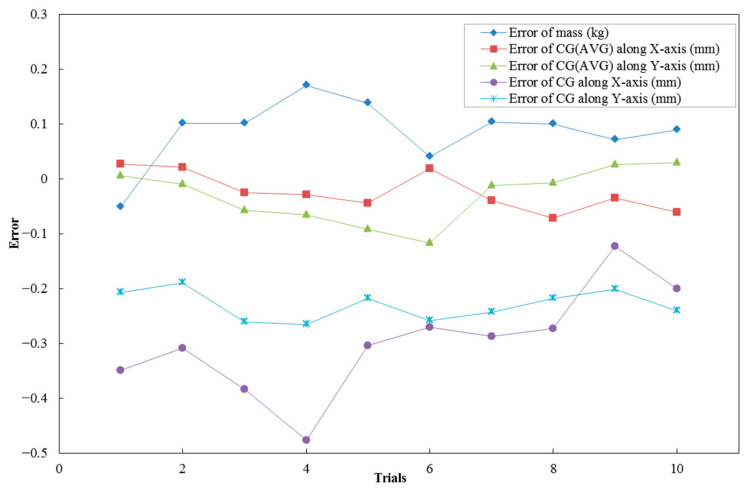
Standard sample mass centroid measurement error.

The standard part was placed on the measuring table at 10 different positions, and the above operation was repeated; the results are shown in Figure 7.

It can be seen that for the mass and centroid measurement system, the error (2σ) of the mass parameter was not greater than 0.2 kg and the repeatability accuracy of the mass measurement can reach 0.02%. The mean value of the centroid measurement results of the four positions was significantly lower than the error of the single position measurement results, and the error uncertainty (2σ) of the centroid measurement was within 0.1 mm.

### 3.2. Example

**Figure 8:**
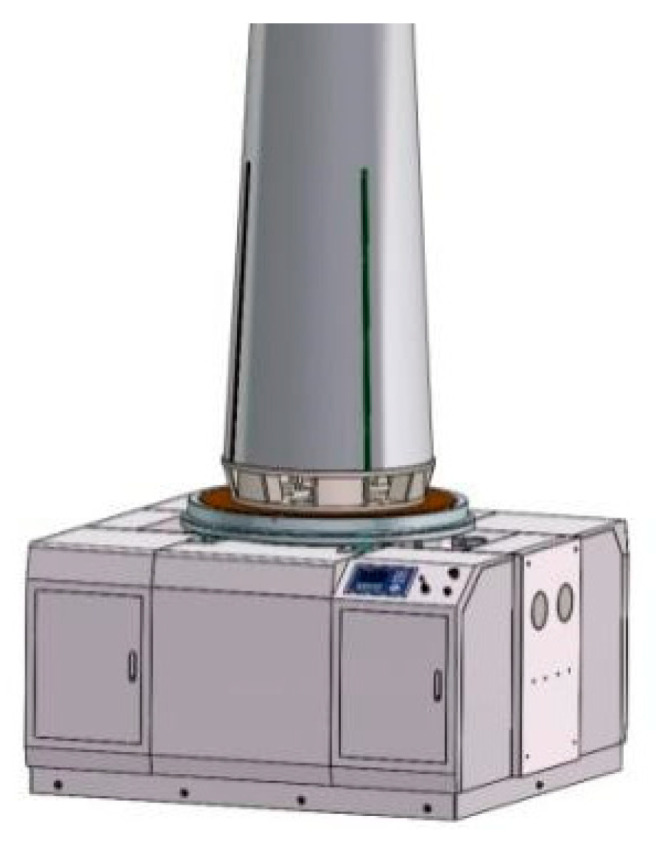
Aircraft mass centroid measurement diagram.

According to the measurement method described in the previous section, a large-sized aircraft with a mass of about 250 kg is used as the object to be measured, and the vertical measurement pose is adopted, as shown in Figure 8. The measured parameters are the mass of the aircraft and its radial two-dimensional centroid, ten sets of experimental cycles were completed, and the results are shown in Table 2. The data of centroid measurement are the averages of multi-station measurement and single-station (station I) measurement, respectively.

It can be seen that the error (2*σ*) of the mass parameter is no more than 0.1 kg, and the repeatability accuracy of the mass parameter is no more than 0.03%. In terms of the 2D centroid, the deviation of the multi-station measurement results is significantly lower than that of traditional single station measurement.

### 3.3. Uncertainty Evaluation of 2D Centroid

Take full scale (3000 kg) as an example: the uncertainty of the load cells is 0.01% (C6); the length design value of sensor group is 1600 mm; each load cell bears the same weight; and the distance L from the center of mass to the tail of the aircraft is about 2000 mm.

(1) Influence of Uncertainty of Load Cell on *Y_CG_*

Differentiate Equation (4) with respect to *Y_CG_*, namely:

(23) $$\Delta y_{1}=\sqrt{\frac{4\left(\partial \Delta P_{1}{}^{2}/\partial y_{\mathrm{cg}}\right)y_{s1}^{2}}{{\left(4\Delta P_{1}\right)}^{2}}}=0.03\mathrm{mm}$$

(2) The influence of the uncertainty of sensor coordinates on *Y_CG_*.

In fact, the laser tracker determines the coordinates of the sensors, and the distance between the tracker and the load cells is less than 5 m; thus, Δ*y*_2_ can be regarded as 0.1 mm.

(3) The influence of the uncertainty of inclination angle of the aircraft on *Y_CG_*. The inclination angle can be controlled at 10″, then Δ*y*_3_ = *Lsin*10″ = 0.1 mm.

(4) The influence of other factors on the *Y_CG_* Δ*y*_4_ = 0.05 mm.

The uncertainty of 2D centroid single-station measurement is:

(24) $$\sigma_{y}=\sqrt{\Delta y_{1}^{2}+\Delta y_{2}^{2}+\Delta y_{3}^{2}+\Delta y_{4}^{2}}=0.15\mathrm{mm}$$

Reasoning from Section 2.4, the multi-station measurement virtually eliminates the effects of inclination angle; thus, the uncertainty is:

(25) $$\sigma_{avg}=\sqrt{\Delta y_{1}^{2}+\Delta y_{2}^{2}+\Delta y_{3}^{2}+\Delta y_{4}^{2}}=0.12\mathrm{mm}$$

According to Equations (24) and (25), it can be seen that the uncertainty of the multi-station measurement is significantly better than that of the single-station measurement.

### 3.4. Comparison and Analysis

As shown in Table 3, this method is compared with the centroid measurement equipment with error compensation proposed in cite [10] and cite [11].

It can be seen that, in terms of standard deviation, the cite [11] is almost an approach to the multi-station method proposed in this paper; however, the shape of the tested aircraft is limited to the rotary structure that the equipment in cite [11] can be compatible with. Although equipment in cite [10] is compatible with aircrafts of different shapes and structures, the standard deviation is still greater than that of the multi-station method proposed in this paper even after compensation. Additionally, the cite [11] relies heavily on the use of laser trackers during the measurement process.

In summary, the self-compensating multi-station centroid measurement method mentioned in this paper can adapt to different shapes of aircraft to the greatest extent while maintaining high measurement accuracy.

## 4. Discussion

In view of the fact that there is little research on the error compensation method of large mass and centroid measurement equipment, this paper proposed an idea of self-compensating multi-station measurement, designed and implemented a multi-station switching device, and optimized the original mass and centroid measurement equipment by combining the principle of high precision measurement. The kinematic model was established for the mechanical structure of the measurement equipment, and the influence of the measured object’s pose error on the measurement results was analyzed. Through theoretical and experimental proofs, the multi-station measurement idea proved that the measurement accuracy after self-compensation was significantly improved compared with that before. At the same time, the solution also has the significant advantages of requiring less product installation, high measurement accuracy, and strong compatibility.

The mass and centroid measurement equipment is theoretically applicable to objects weighing 3000 kg; since the measured object is not limited to a single outline structure, it can also be considered to extend its application to other fields besides aircraft.

## Figures and Tables

**Table 1:** Specification and design requirements of the systems.

Item	Dimensions
Test parameters	Mass; CG along axes X, Y, and Z;
Mass measurement range	100–3000 kg
Length measurement range	800–4000 mm
Mass measurement accuracy	≤0.03%
Radial CG measurement accuracy	≤±0.15 mm
Applicable product maximum diameter	No less than φ 1650 mm

**Table 2:** Measurement results of mass and centroid.

Test No.	Multi-Station Coordinate of CG (mm)		Station I Coordinate of CG (mm)		Mass (kg)
	Y	Z	Y	Z	
1	28.120	−4.056	27.771	−4.263	258.529
2	28.038	−4.073	27.730	−4.262	258.632
3	28.022	−4.059	27.638	−4.320	258.632
4	28.044	−4.079	27.567	−4.345	258.641
5	28.012	−4.098	27.708	−4.316	258.628
6	28.016	−4.090	27.746	−4.348	258.621
7	28.022	−4.070	27.736	−4.313	258.640
8	27.987	−4.042	27.714	−4.260	258.680
9	28.001	−3.975	27.878	−4.176	258.652
10	27.993	−4.046	27.793	−4.287	258.670
Average result	28.025	−4.059	27.728	−4.289	258.633
Standard deviation	0.036	0.033	0.080	0.049	0.038
Repeatability	—	—	—	—	0.014%

**Table 3:** Comparison of 3 self-compensating centroid measuring equipment.

	Multi-Station Equipment	Equipment in Cite [10]	Equipment in Cite [11]
Standard deviation	0.12 mm	0.18 mm	0.14 mm
Applicable scope of aircraft	Rotary body and irregular body	Rotary body and irregular body	Rotary body

## Data Availability

Not applicable.
